# Development and Psychometric Validation of Mental Health Placement Readiness Questionnaire (Mental Health PEPR‐Q10) for Nursing and Midwifery Students

**DOI:** 10.1155/nrp/4210180

**Published:** 2026-11-10

**Authors:** Caglayan Yasan, Alan Hayes, Abdi Osman, Jahar Bhowmik

**Affiliations:** ^1^ Discipline of Nursing and Midwifery College of Sports, Health, and Engineering Victoria University Melbourne Australia vu.edu.au; ^2^ Institute for Health and Sport Victoria University Melbourne Australia vu.edu.au; ^3^ Department of Biomedical, Health and Exercise Sciences Swinburne University of Technology Melbourne Victoria Australia swinburne.edu.au

**Keywords:** development, mental health clinical readiness, nursing and midwifery student placement, survey, validation

## Abstract

**Aim:**

This study developed and validated a 10‐item Mental Health Professional Experience Readiness Questionnaire (Mental Health‐PEPR‐Q10) to assess mental health placement in nursing and nursing/midwifery dual degree students. It also examined students’ self‐perceived preparedness for placement following real‐life actors’ simulations.

**Background:**

No validated tool currently exists to evaluate the student’s readiness for mental health placements. This study addresses practical skills, self‐efficacy and knowledge acquisition in Australian nursing education.

**Method:**

A methodological cross‐sectional survey design was used. Of 510 eligible second‐year nursing and third‐year nursing/midwifery dual‐degree students, 232 completed the questionnaire (45.5% response rate). The authors developed the 10 item‐Mental Health PEPR‐Q10 to assess practical skills, self‐efficacy following simulation with real‐life actors and Objective Structured Clinical Assessment, and mental health knowledge. Responses were recorded using a 5‐point Likert scale. Principal component and factor analysis evaluated and validated the scale’s psychometric properties.

**Results:**

A single‐factor solution of exploratory factor analysis explained 74.41% of the total variance. The 10‐item Mental Health PEPR‐Q10 scale supports its factorial component and reliability with Cronbach’ alpha of 0.964. The fit indices from the single‐factor confirmatory factor analysis suggest a good model fit, with a standardised root mean square residual (SRMR) of = 0.047, comparative fit index (CFI) = 0.962, normal fit index (NFI) = 0.927 and root mean square error (RMSE) = 0.06.

**Conclusion:**

The Mental Health‐PEPR‐Q10 is a robust, validated tool for assessing nursing and nursing/midwifery students self‐perceived mental health placement readiness in Australia, providing a foundation for the future evaluation of simulation‐based preparation.

**Summary**
• What is already known about this topic?
◦ Simulation is widely used to support the development of nursing students’ mental health Professional Experience Placement (PEP) skills and knowledge.
• What does this article add?
◦ This study provides a validated 10‐item Mental Health Professional Experience Placement Readiness Questionnaire (Mental Health PEPR‐Q10) to assess nursing and nursing/midwifery dual degree student readiness for mental health PEP.
◦ This tool facilitates further research into the Mental Health PEP of undergraduate nursing and nursing/midwifery dual degree education programs which will ultimately contribute to the preparation of graduates.
◦ Students perceived real‐life actor simulations as helpful in preparing nursing and nursing/midwifery students for mental health PEP.
◦ Well‐prepared graduates will improve patient care outcomes within the mental health setting.

## 1. Introduction

In nursing education, mental health PEPs are integral for student growth and enhancing the overall wellbeing of the community. Australia is projected to face a significant shortfall of 87,645 mental health professionals by 2030, a significant increase from earlier estimates [1]. Concurrently, the World Health Organisation [2] anticipates a global deficit of 4.5 million nurses by 2030, highlighting the imminent need for additional trained mental health nurses within the next five years. PEP provides nursing students with invaluable opportunities to engage with patients under the supervision of qualified healthcare professionals, thereby strengthening clinical and practical skills while fostering the development of professional identity as a mental health nurse [3]. However, PEP requirements vary internationally: Australia mandates a minimum of 800 h [4], New Zealand requires 1100 h, and United Kingdom requires 2300 h [5]. Increasing student enrolments and supervisor’s workloads continue to present ongoing challenges.

Students frequently report heightened anxiety before commencing PEP, due to preconceived notions, limited exposure to mental health consumers and stigma surrounding mental illness [6]. This anxiety, coupled with scarcity of mental health PEP, impedes the development of essential mental health nursing skills. As the demand for trauma‐informed care (TIC), strength‐based interventions and recovery‐focused approaches increases, such barriers may hinder students’ ability to establish therapeutic relationships with mental health patients and may result in avoidant behaviours [7, 8]. Despite the importance of these competencies (Lim et al. [9]), no standardised tools currently exist to assess students’ readiness. Unlike general clinical competence (the ability to perform tasks, procedures and techniques) or self‐confidence alone, placement readiness is a multidimensional construct that integrates professional skills, communication, self‐management and self‐confidence, applied within a specific clinical context [10].

While no universal standardised tool exists for mental health professionals, several validated tools are used within related fields. For example, the Australian Nursing Standards Assessment Tool is a validated assessment tool that measures performance against Nursing and Midwifery Board of Australian registered nurse standards for practice during clinical placements [11] but does not address mental health conditions, TIC, strength‐based practices, clinical deterioration or mental state examination and risk assessment. Similarly, the Casey Fink Graduate Nurse Experience Survey assesses graduate nurses’ readiness, confidence and experience before transitioning to practice [12]. Yet, it lacks specificity for mental health–related competencies. The Clinical Learning Environment, Supervision and Nurse Teacher scale, validated in multiple countries [13], also remains nonspecific to the mental health profession. Adapting or extending these existing measures was considered unsuitable for the present purpose. Each was designed to evaluate general clinical performance, readiness to transition into qualified practice or the quality of clinical learning environment, rather than preplacement readiness and none captures the mental health‐specific competencies. Therefore, a purpose‐built tool was required to assess mental health placement readiness specifically.

To address this identified gap in mental health PEP skills, academics at Victoria University have integrated trained real‐life actors into simulation and Objective Structured Clinical Assessment (OSCA) sessions. This approach is intended to enhance students’ understanding and application of various mental health conditions, TIC, strength‐based practices, clinical deterioration and conducting mental state examination and risk assessment. The objective of this study is (1) to develop and validate a concise 10‐item Mental Health Professional Experience Placement Readiness Questionnaire (Mental Health PEPR‐Q10) as a readiness assessment tool, (2) to assess the level of readiness among nursing and nursing/midwifery dual degree students preparing for mental health PEP within a Block Model framework and (3) to assess the students’ perceived preparedness for mental health PEP following real‐life actor simulation and OSCA. The Block Model enables students to focus intensively on one unit at a time over 4‐week blocks, in contrast to the traditional semester mode [14].

## 2. Methods

### 2.1. Study Design

A methodological research design was employed to collect data using an online cross‐sectional survey to determine the nursing and midwifery student’s readiness for Mental Health PEP through the developed and validation of the 10 item‐Mental Health PEPR‐Q10. This approach was chosen as it focuses on developing, validating and evaluating research tools, thereby ensuring the reliability and validity of the findings [15].

### 2.2. Study Sample, Setting and Recruitment

The research study was conducted at Victoria University in Melbourne from January to June 2024. A convenient sampling method was employed targeting to recruit 510 second‐year nursing and third‐year dual degree nursing/midwifery students enrolled in the HNB2002 Mental Health Unit. A total of 232 students completed the survey, resulting in a response rate of 45.9%.

In Australia, nursing and midwifery are distinct regulated professions with separate registration standards. However, the dual‐degree program at Victoria University is designed so that students complete both the full nursing and midwifery curricula, including shared mental health units such as HNB2002. These dual‐degree students undertake the same mental health nursing content and assessments as single‐degree nursing students. Therefore, both groups were exposed to equivalent mental health education relevant to this study. No significant differences in prior mental health curriculum exposure were anticipated between the two groups at the time of data collection.

On the first day of the unit, participants received an invitation via the institutional learning management system (Collaborate) through the Mental Health Unit News Feed by an individual not involved in teaching nursing units, to reduce coercion. Additional information was disseminated through flyers and group emails. Participants had the options to complete the survey by clicking a link in the group email or scan a QR code from flyers posted outside the OSCA rooms and/or handed by facilitators after the OSCA sessions. Participation was voluntary, with no coercion and the option to withdraw at any point.

### 2.3. Inclusion and Exclusion

All second‐year nursing and third‐year dual degree nursing/midwifery students enrolled in the HNB2002 Mental Health unit (Contemporary Nursing and Mental Health A) at Victoria University, Melbourne, during the study period (Jan–Jun 24) were eligible.

No exclusion criteria were applied. This approach ensured inclusion of entire target cohort, consistent with the convenience sampling method, and minimising the risk of bias.

### 2.4. Data Collection, Measures and Instruments

Data were collected online via the QualtricsXM platform after students completed the OSCA. The survey included 10 questions on a 5‐point Likert scale (from strongly disagree to strongly agree) to assess students’ readiness for PEP, as well as on demographic characteristics. The questionnaire was developed by the research team in alignment with the research questions and the learning outcomes of the Mental Health unit. The Mental Health PEPR‐Q10 was developed as a purpose‐built, concise measure of students’ self‐perceived readiness for mental health PEP. Item generation was theory‐ and curriculum‐driven: Items were derived from the learning outcomes of the HNB2002 Mental Health unit and from contemporary literature on the use of real‐life actors (standardised patients) in mental health simulation and OSCA, with emphasis on TIC, strength‐based practice, therapeutic communication, mental state and risk assessment, recognition of clinical deterioration and person‐centred care. Ten items were developed to directly map onto these domains and were reviewed by experts in the field. The tool was intentionally designed with exact 10 items to capture the mental health readiness constructs; no additional items were generated for subsequent removal, and all 10 items were retained through validation, as supported by their factor loading and communalities. There were no negatively worded items among the 10 items in the questionnaire; therefore, reverse coding was not required for the analysis. To establish content validity and ensure relevance to clinical placement, input was sought from two mental health nursing experts, three registered nurses (including one with extensive mental health experience) and one physiotherapist. This collaborative expert‐panel approach is a recognised method for achieving content validity when developing instruments in nursing education and simulation research [16–18]. Expert input was collated qualitatively, and items were refined by consensus to improve clarity and relevance before collecting data. A formal quantitative content validity index (CVI) was calculated (Table 1) based on six experts opinion using the formula, Item‐Level Content Validity Index(I‐CVI) = (Number of experts rating the item as relevant)/N, and CVR = (ne−N/2)/(N/2), where one is the number of experts rating an item as essential and N is the total number of experts. In addition, before administering the questionnaire, it was pretested by the members of the research team who developed, reviewed and completed the items tool to check their wording for clarity. No separate pilot tests or cognitive interviews were conducted with students. Preadministration refinement therefore relied on this pretesting by the research team and the expert‐panel content review described above.

**TABLE 1:** Item‐Level Content Validity Index (I‐CVI) and content validity ratio (CVR) of the 10‐item questionnaire.

Item	Experts rating relevant/strongly agree	I‐CVI	Experts rating “essential”	CVR
Item 1	6/6	1.00	6/6	1.00
Item 2	6/6	1.00	6/6	1.00
Item 3	6/6	1.00	6/6	1.00
Item 4	6/6	1.00	6/6	1.00
Item 5	6/6	1.00	6/6	1.00
Item 6	6/6	1.00	6/6	1.00
Item 7	6/6	1.00	6/6	1.00
Item 8	6/6	1.00	6/6	1.00
Item 9	6/6	1.00	6/6	1.00
Item 10	6/6	1.00	6/6	1.00

In the absence of existing validated instruments specifically addressing students’ readiness for mental health PEP, the questionnaire was informed by key principles in contemporary literature on the use of real‐life actors (also known as standardised patients) in mental health simulation and OSCA and their perceived transferability to PEP. In particular, it highlighted the significance of TIC and strength‐based interventions in mental health nursing education and practice [19, 20].

Simulation with real‐life actors has been reported in the literature as a pedagogical strategy for preparing undergraduate nursing students for mental health PEP. It has been associated with therapeutic communication, understanding of mental health symptoms, mental state examination and risk assessment, recognition of clinical deterioration, person‐centred care and overall confidence and preparedness for PEP [6, 8, 21, 22].

The mental health unit curriculum also covers comprehensive mental state examination and risk assessment during simulation, recognition of patient deterioration through verbal and nonverbal communication and the delivery of patient‐centred care. Therefore, the questionnaire was designed to evaluate students’ perceptions of the simulation and OSCA experience, especially the use of real‐life actors and their perceived contribution to the acquisition of these specific skills and knowledge and their transferability to real‐world PEP.

### 2.5. Statistical Analysis

Data were obtained on a sample of 232 participants. Initial data screening was conducted to identify any unusual patterns or missing values across variables. No missing data were observed for the 10 items. Descriptive statistics were conducted to describe and explore the sample (Table 2). The distribution of participants’ responses to individual Mental Health PEPR‐Q10 items, measured on a 5‐point Likert scale (strongly disagree to strongly agree), was examined to assess the sensitivity of the scale (Table 3). Factor analysis and item‐reduction techniques were then used to develop and validate the scale based on responses to 10 items questionnaire.

**TABLE 2:** Sociodemographic characteristics of the participants in the survey (*N* = 232).

Variable	N/Mean^∗^	Percentage/SD^∗^
Gender		
Male	23	9.9
Female	205	88.4
Prefer not to say	4	1.7
Residential area (area of living)		
Metropolitan	216	93.1
Regional	16	6.9
Degree currently enrolled in		
Bachelor of Nursing	204	87.9
Dual Degree of Midwifery/Nursing	28	12.1
Year level currently enrolled in		
Year 2 Nursing	206	88.8
Year 3 Midwifery	26	11.2
Current working area		
Health care	98	42.2
Other	134	57.8
Working as a division two enrolled nurse		
Yes	68	29.3
No	164	70.7
Age (in years)^∗^	26.2	7.9

^∗^Metric variables.

**TABLE 3:** Distribution of participants’ opinion on the individual items of the Mental Health PEPR‐Q10 (*n* = 232).

Items/Questions	Strongly disagree	Disagree	Neither disagree nor agree	Agree	Strongly agree	Mean (SD)
	*n* (%)					
1. You feel confident going out into clinical	0	6	15	117	94	4.29
placement having completed the three simulation sessions and OSCA	(0)	(2.6)	(6.5)	(50.4)	(40.5)	(0.70)
2. The simulations with real‐life actors and	0	0	1	74	157	4.67
OSCA has influenced your growth and development as a professional nursing or midwifery student	(0)	(0)	(0.4)	(31.9)	(67.7)	(0.48)
3. The real‐life actors during simulation and	0	2	1	73	156	4.65
OSCA has given you the set of skills and knowledge to prepare for clinical placement	(0)	(0.9)	(0.4)	(31.5)	(67.2)	(0.54)
4. The real‐life actors during simulation	0	0	0	73	159	4.69
prepared you to better understand the symptoms of mental health illness/disorders in clinical placement	(0)	(0)	(0)	(31.5)	(68.5)	(0.47)
5. Having real‐life actors during simulation	0	0	1	68	163	4.69
and OSCA influenced your therapeutic verbal and non‐verbal communication skills in preparing for clinical placement	(0)	(0)	(0.4)	(29.3)	(70.3)	(0.47)
6. Having real‐life actors in your simulation	0	2	3	68	159	4.65
and OSCA prepared you to provide person‐centred care on clinical placement	(0)	(0.9)	(1.3)	(29.3)	(68.5)	(0.55)
7. Having real‐life actors in your simulation	0	2	14	85	131	4.69
and OSCA provided you with adequate skills and knowledge to provide trauma‐informed care on clinical placements	(0)	(0.9)	(6.0)	(36.6)	(56.5)	(0.69)
8. The real‐life actors in your simulation and	0	2	9	85	136	4.51
OSCA provided you with adequate skills and knowledge to provide strength‐based practice on clinical placements	(0)	(0.9)	(3.9)	(36.6)	(58.6)	(0.66)
9. The real‐life actors in your simulations	0	2	6	88	136	4.54
and OSCA provided you with skills and knowledge to recognise clinical deterioration in patients with mental health illness/disorders in clinical placement	(0)	(0.9)	(2.6)	(37.9)	(58.6)	(0.59)
10. Completion of simulation and OSCA	0	1	6	82	143	4.58
with real‐life actors prepared you in conducting a mental state and a risk assessment on a real patient in a clinical placement	(0)	(0.4)	(2.6)	(35.3)	(61.6)	(0.57)

Abbreviation: OSCA = Objective Structured Clinical Assessment.

Exploratory factor analysis (EFA), principal component analysis, Pearson correlations, confirmatory factor analysis (CFA) and Cronbach’s alpha were used to assess the reliability and validity of the Mental Health PEPR‐Q10 scale. The analyses of psychometric properties were based on the sum of the total Mental Health PEPR‐Q10 scores, giving a wider distribution of readiness scores. The psychometric properties of the developed Mental Health PEPR‐Q10 questionnaire were analysed according to the following criteria for measurement and psychometric properties [23–25] by randomly splitting the total sample of 232 data into roughly equal halves. After a random split, a sample of 118 was assigned to Group 1 for EFA, while the remaining 114 participants were allocated to Group 2 for CFA. A sample size of 118 participants was sufficient to conduct the EFA, while the remaining 114 participants were used for the CFA with 10 items [26], providing adequate statistical power at the 5% level of significance (Hair et al. [27]). To determine the construct validity of the Mental Health PEPR‐Q10 initially a principal component analysis with an Oblimin rotation was performed on all 10 items, as suggested in other validation studies [28]. However, to improve the validity measure by reducing residuals finally EFA was performed using principal axis factoring extraction method, considering as relevant in our set of data as it does not require normally distributed data [29]. Oblique Promax rotation method was used as it permits correlation of the factors [30]. Factor loading and communalities were used in validating contribution of each of the 10 items in the scale. Prior to factor extraction, we assessed sampling adequacy and suitability of factor analysis. Kaiser–Meyer–Olkin measure yielded a value of 0.924, indicating excellent suitability for factor analysis. Bartlett’s test of sphericity was statistically significant (*χ* ^2^ = 1417.31, df = 45, *p* < 0.001), confirming that the correlation matrix contained sufficient interitem relationship to proceed. Model fit was assessed using recommended indices, including a root mean square error of approximation below 0.07, standardised root mean square residual (SRMR) below 0.08 and a comparative fit index (CFI) or Tucker–Lewis index greater than 0.95 [31]. The significance of the mediation effect was assessed based on a bootstrap analysis of 5000 samples. For CFA, model modifications were considered only when supported by theoretical and substantive considerations. Modification indices were examined to identify potential sources of model misfit; however, parameters were added or freed only where there was a clear conceptual justification and where the modification was consistent with the underlying construct and item content. The aim was to improve model fit without compromising the theoretical structure or interpretability of the measurement model. Data were analysed using SPSS and AMOS Version 30.

### 2.6. Ethical Considerations

The research received ethics approval from the Victoria University Human Research Ethics Committee (approval number: HRE23‐165). Participant and consent information and the survey were all administered electronically using Qualtrics. Participants were provided with participation information and advised to proceed to the survey if they consent to participate.

## 3. Results

A total of 232 students completed the online readiness survey, including 204 Bachelor of Nursing students and 28 dual degree Midwifery/Nursing students. Table 2 presents the demographics of the sample and shows that 88.8% (*n* = 206) were second‐year nursing students, while 11.2% (*n* = 26) were third‐year midwifery students. Most participants identified as female (88.4%), with 11.2% male, and 1.3% (*n* = 4) preferring not to disclose their gender. The average age of students was 26.2 years (SD = 7.9), ranging from 19 to 55 years. Among respondents, 29.3% (*n* = 68) were Division 2 nurses, while 70.7% (*n* = 164) were not. Additionally, 42.2% (*n* = 98) worked in the healthcare sector, with the remaining 57.8% (*n* = 134) employed in other sectors.

Table 3 presents the distribution of participants’ opinions on the individual items of the Mental Health PEPR‐Q10. The results indicate that over 80% of respondents agreed that the use of real‐life actors during simulation and OSCA’s was useful in enhancing their readiness for clinical placement. A relatively high proportion of “strongly agree” responses was observed for most questions (Mental Health PEPR‐Q10), reflecting participants’ assessment of their acquired skills and knowledge for clinical placement upon completing their studies. As shown in Table 4, the majority of items in the Mental Health PEPR‐Q10 instrument demonstrated significant correlations at *p* < 0.01, supporting the internal consistency of the items and validating their suitability for EFA.

**TABLE 4:** Pearson correlation analysis of the items (*n* = 118).

	1	2	3	4	5	6	7	8	9	10
1	1.000									
2	0.620^∗∗^	1.000								
3	0.717^∗∗^	0.724^∗∗^	1.000							
4	0.587^∗∗^	0.798^∗∗^	0.799^∗∗^	1.000						
5	0.534^∗∗^	0.751^∗∗^	0.730^∗∗^	0.737^∗∗^	1.000					
6	0.660^∗∗^	0.732^∗∗^	0.798^∗∗^	0.725^∗∗^	0.793^∗∗^	1.000				
7	0.620^∗∗^	0.712^∗∗^	0.668^∗∗^	0.704^∗∗^	0.673^∗∗^	0.709^∗∗^	1.000			
8	0.660^∗∗^	0.712^∗∗^	0.743^∗∗^	0.720^∗∗^	0.671^∗∗^	0.798^∗∗^	0.861^∗∗^	1.000		
9	0.699^∗∗^	0.703^∗∗^	0.712^∗∗^	0.685^∗∗^	0.712^∗∗^	0.786^∗∗^	0.797^∗∗^	0.853^∗∗^	1.000	
10	0.557^∗∗^	0.668^∗∗^	0.703^∗∗^	0.673^∗∗^	0.681^∗∗^	0.743^∗∗^	0.671^∗∗^	0.707^∗∗^	0.790^∗∗^	1.000

^∗∗^Correlation is significant at the 0.01 level (2‐tailed).

Principal component analysis and factor analysis on the data (*n* = 118) indicated a single factor for the 10 readiness questions (Figure 1), with this factor explaining 74.41% of the variance and all item communalities above 0.5. Reliability analysis also demonstrated strong internal consistency, with a Cronbach’s alpha of 0.964 (95% CI: 0.953–0.973), item variances above one, and all squared multiple correlation coefficients exceeding 0.5. Item deletions did not enhance internal consistency for any items. A summated score for the factor “Readiness” was created for further analysis with score ranging from 10 to 50 where higher score means better readiness knowledge.

**FIGURE 1:**
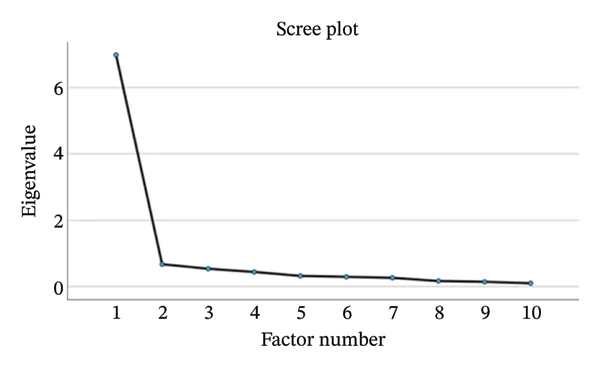
Scree plot of the initial exploratory factor analysis, extracted single factor with Eigenvalue > 1.

The results from the CFA conducted on the second half of the data (*n* = 114) showed acceptable to good model fit. Although the chi‐square test was statistically significant (*X* ^2^(31) = 63.87, *p* < 0.001), suggesting the model may not fit the data perfectly, other fit indices indicated an acceptable fit overall. The CFI of 0.962, the Normed Fit Index (NFI) of 0.927, the root mean square error of approximation (RMSEA) of 0.060 (95% CI: 0.044–0.071) and a SRMR of 0.047, collectively suggesting a reasonably good fit [31]. Convergent validity was supported by significant factor loadings (Table 5) and acceptable average variance extracted (AVE) values of for the factor Readiness with AVE = 0.51. Detailed information on the fitted CFA model, including standardised factor loadings, residuals, factor correlations and modification decisions, is presented in Figure 2. Multicollinearity was not an issue, with all variance inflation factor (VIF) values below 5. No notable floor or ceiling effects were observed, indicating that the scale adequately captured the full range of participant responses.

**TABLE 5:** Factor loading and communities (both initial and extracted) from exploratory factor analysis for one‐factor solution, eigenvalue and extracted percentage of variance, Kaiser–Meyer–Olkin and reliability analysis (Cronbach’s alpha) (*n* = 118).

Items	Factor loadings	Communalities	
		Initial extracted	
1. You feel confident going out into clinical placement having completed the three simulation sessions and OSCA	0.718	0.578	0.515
2. The simulations with real‐life actors and OSCA has influenced your growth and development as a professional nursing or midwifery student	0.823	0.716	0.677
3. The real‐life actors during simulation and OSCA has given you the set of skills and knowledge to prepare for clinical placement	0.914	0.839	0.835
4. The real‐life actors during simulation prepared you to better understand the symptoms of mental health illness/disorders in clinical placement	0.846	0.783	0.716
5. Having real‐life actors during simulation and OSCA influenced your therapeutic verbal and non‐verbal communication skills in preparing for clinical placement	0.827	0.778	0.684
6. Having real‐life actors in your simulation and OSCA prepared you to provide person‐centred care on clinical placement	0.941	0.880	0.885
7. The simulation and OSCA have provided you with adequate skills and knowledge to provide trauma‐informed care on clinical placements	0.861	0.841	0.741
8. The simulation and OSCA have provided you with adequate skills and knowledge to provide strength‐based practice on clinical placements	0.911	0.912	0.831
9. The simulations and OSCA have provided you with skills and knowledge to recognise clinical deterioration in patients with mental health illness/disorders in clinical placement	0.920	0.876	0.847
10. Completion of simulation and OSCA prepared you in conducting a mental state and a risk assessment on a real patient in a clinical placement	0.844	0.787	0.712
Eigenvalue (extracted)	7.68		
Percentage of variance explained (extracted)	74.41		
Kaiser–Meyer–Olkin measure of sample adequacy	0.924		
Cronbach’s alpha(*α*)–measure of reliability	0.95		

Abbreviation: OSCA = Objective Structured Clinical Assessment.

**FIGURE 2:**
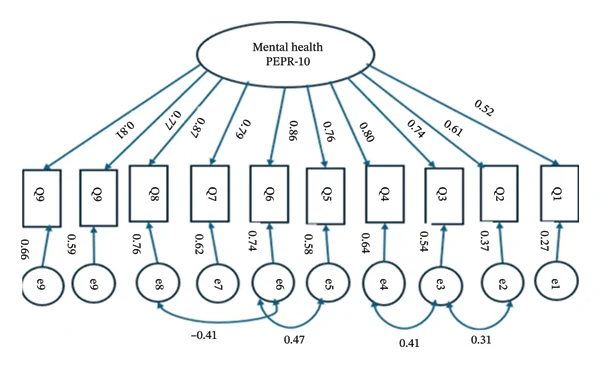
A single‐factor fitted model in CFA (*n* = 114).

The findings from the expert evaluation of the 10‐item scale are presented in Table 1. All 10 items demonstrated excellent content validity, with an I‐CVI of 1.00 and a CVR of 1.00 for each item, indicating unanimous agreement among the six expert researchers regarding the relevance and essentiality of all questionnaire items.

## 4. Discussion

The primary finding of this study is that the 10‐item Mental Health PEPR‐Q10 is a statistically validated instrument with strong internal consistency, a single‐factor structure explaining 74.41% of the variance and suitability for assessing nursing and midwifery students’ readiness for mental health PEP in Australia. As the first validated tool specially designed for this purpose, the Mental Health PEPR‐10 addresses a significant gap in the literature, where no comparable instrument previously existed for direct comparison. This validation enables reliable placement assessment of student preparedness, potentially informing tailored support strategies to enrich the PEP experience. It should be emphasised that this study establishes the psychometric properties of the Mental health PEPR‐Q10 as a measure of students’ self‐perceived readiness. It was not designed to demonstrate the educational effectiveness of real‐life actor simulation, which would require a controlled or longitudinal design.

The psychometric performance of the Mental Health PEPR‐Q10 compares favourably with established readiness and simulation‐related self‐efficacy instruments used in nursing education. While existing measures such as the Casey–Fink Readiness for Practice Survey assess broader perceptions of readiness for professional practice, and newer instruments such as the Nursing Student Self‐Efficacy in Clinical Skills Scale focus on confidence in performing core clinical skills, the PEPR‐Q10 extends this literature by targeting perceived preparedness specifically within mental health nursing education [12, 32].

PEPs are crucial in shaping nursing students’ career decision‐making decisions and facilitating the transition from the academia to clinical practice [33]. The present study found that students reported feeling confident entering mental health PEP following three simulation sessions and an OSCA involving real‐life actors. These results align with existing evidence that simulation‐based education reduces stress, builds self‐efficacy and associated with greater perceived preparedness for both OSCA and subsequent mental health PEP (Hebel et al. [34]).

Regulatory bodies and healthcare workplaces require nursing education to equip nursing students with the competencies required for effective practice upon graduation [35]. The current findings indicate students perceived simulation and OSCA are helpful preparatory strategies for key mental health skills, including mental state examination and risk assessment and recognising clinical deterioration in patients with mental health illnesses. These competencies are essential for timely referrals and improved patient outcomes, as supported by evidence that proficiency in conducting MSE equips nurses to identify deterioration [36]. Recent literature further reinforces MSE as a core competency in mental health nursing education and practice internationally (Volvevska et al. [37]), which informed the inclusion of related questions in the Mental Health PEPR‐Q10.

Contemporary mental health services increasingly emphasise TIC practices to support recovery, minimise retraumatisation and foster collaborative, empowering care environment [38]. Systematic review highlights the value of experiential training over didactic methods to build practical skills in areas of trauma‐informed approaches, mental state examination and risk assessment and strengths‐based interventions [39]. The validated PERP‐10 responds to this need by providing an accurate measure of students’ perceived readiness, which may help clarify expectations, and identify individual strengths and areas requiring support prior to PEP.

For educational institutions, the tool offers a means of monitoring student‐reported readiness that may inform curriculum refinement and contribute to accreditation and quality assurance processes. It may also strengthen partnership with placement providers by documenting students’ self‐reported preparedness, fostering greater confidence among supervisors. Overall, the 10‐item Mental Health PEPR‐Q10 provides a reliable, single‐factor instrument for assessing readiness with the summated “Readiness” offering potential utility for benchmarking against university assessments and future workplace performance.

## 5. Strength and Limitations

The primary strength of this study lies in the development of the Mental Health PEPR‐Q10 scale, where indicators for testing discriminant and concurrent predictive validity of the scores yielded expected results. Additionally, the survey demonstrated that the instrument performed well in assessing the Mental Health PEPR of nursing and nursing/midwifery dual degree students. These findings can guide and support political and professional decision‐making aimed at enhancing Mental Health PEPR before students transition to professional practice.

This study has some limitations. Although the 45.9% response rate exceeds typical rates for student surveys, the possibility of nonresponse bias cannot be excluded. In addition, participation was voluntary and depended on students opting in, introducing the potential for self‐selection bias, whereby students who held more positive or confident views of the simulation experience may have been more inclined to respond and thereby inflate readiness scores. The sample was drawn exclusively from one Australian university, which may limit broader generalisability. Recruitment at a single institute means findings reflect one curriculum, cohort profile and simulation model (real‐life actors delivered within the Block Model) and may not transfer to programs with different educational structures, student demographics or placement contexts. Future validation involving larger, multi‐institutional cohorts recruited across multiple universities and diverse healthcare and placement settings, ideally including international sites, is required before claims of broader national or international applicability can be made and to establish the external validity and cross‐contextual robustness of the Mental Health PEPR‐Q10. Additionally, the cross‐sectional design precludes causal inferences regarding the impact of real‐life actor simulations on placement readiness. However, the internal consistency and the single factor contributing to two‐thirds of the variance suggest that this would not be a limiting factor in use of the survey. Future research could also focus on the specific Mental Health PEPR needs of smaller subgroups, such as culturally and linguistically diverse communities. From a statistical standpoint, one limitation of the data was the lack of responses in the ‘Strongly disagree’ category and the infrequent selection of ‘Disagree’, which could impact the variability of responses. A limitation of this study is that, although CFA was used to evaluate psychometric properties, the analysis relied on splitting the sample of 232 participants into roughly equal halves, which may reduce the robustness of the findings due to the smaller subgroup sample sizes. Another limitation of this study is that test–retest reliability was not assessed, and therefore the temporal stability of the scale could not be established. The high level of agreement observed across the 5‐point Likert‐scale items may have resulted in a potential ceiling effect, whereby limited variability in responses could attenuate the ability of the questionnaire to distinguish between participants with higher levels of the underlying construct. Although no formal ceiling‐effect criteria were applied, this restricted response distribution should be considered when interpreting the reported validity evidence, particularly its ability to discriminate at the upper end of the construct. Lastly, the cross‐sectional design of the study limits the ability to infer causality between factors associated with Mental Health PEPR. In addition, although the questionnaire was pretested by the research team who developed it, it was not pilot tested or subjected to cognitive interviewing with students prior to administration, so students’ understanding of the item wording was not directly assessed. Future studies should include cognitive interviews with students to further support the response‐process validity of the Mental Health PEPR‐Q10.

## 6. Conclusion

A validated tool is needed to monitor and benchmark nursing and dual‐degree nursing/midwifery students’ preparedness for Mental Health PEP. The 10‐item Mental Health PEPR has been validated in an Australian sample of nursing and dual degree nursing/midwifery students and demonstrates acceptable psychometric properties. This study provides the first evidence of its use and establishes the PEPR‐Q10 as the first validated instrument specially designed to measure Mental Health PEP readiness in nursing and dual‐degree nursing/midwifery students in Australia. While the instrument shows promise, further external validation in larger and more representative samples is required before claims of broader national or international applicability can be made. Future research should prioritise longitudinal studies that examine the predictive validity of PEPR‐Q10 scores against actual clinical placement performance, rather than relying solely on perceived readiness. Such exploration will be essential to confirm the tool’s practical utility and to inform educational programs, interventions and policy aimed at strengthening Mental Health PEP preparedness.

## Author Contributions

Contributions to conception and design, acquisition of data, analysis and interpretation of data: Caglayan Yasan, Alan Hayes and Jahar Bhowmik.

Drafting the manuscript or revising it critically for important intellectual content: Caglayan Yasan, Alan Hayes, Abdi Osman and Jahar Bhowmik.

Agreed to be accountable for all aspects of the work in ensuring that questions related to the accuracy or integrity of any part of the work are appropriately investigated and resolved: Caglayan Yasan, Alan Hayes, Abdi Osman and Jahar Bhowmik.

## Funding

This research received no specific grant from any funding agency in the public, commercial or not‐for‐profit sectors.

## Disclosure

All authors gave final approval of the version to be published and participated sufficiently in the work to take public responsibility for appropriate portions of the content.

## Ethics Statement

The research study received ethics approval from the Victoria University Ethics Committee (approval number: HRE23‐165).

## Conflicts of Interest

The authors declare no conflicts of interest.

## Data Availability

The data that support the findings of this study are available on request from the corresponding author. The data are not publicly available due to privacy or ethical restrictions.
